# Safety, Tolerability, and Immunogenicity of a Recombinant Nonavalent Human Papillomavirus Vaccine (*Escherichia coli*) in Healthy Chinese Women Aged 18–45 Years: A Phase 1 Clinical Trial

**DOI:** 10.3390/vaccines13050511

**Published:** 2025-05-13

**Authors:** Mingwei Wei, Weiwei Han, Jing Zhang, Yongjiang Liu, Hongyang Yu, Jingxin Li, Wenjuan Wang

**Affiliations:** 1 School of Public Health, National Vaccine Innovation Platform, Nanjing Medical University, Nanjing 211166, China; js_wmw@163.com (M.W.); hanweiwei9978@163.com (W.H.); 2 Department of Vaccine Clinical Evaluation, Jiangsu Provincial Center for Disease Control and Prevention (Jiangsu Provincial Academy of Preventive Medicine), Nanjing 210009, China; 3 Jiangsu Provincial Medical Innovation Center, National Health Commission (NHC) Key Laboratory of Enteric Pathogenic Microbiology, Jiangsu Provincial Center for Disease Control and Prevention (Jiangsu Provincial Academy of Preventive Medicine), Nanjing 210009, China; 4 Sheyang City Center for Disease Control and Prevention, Yancheng 224499, China; zjsycdc2005@163.com; 5 Beijing Health Guard Biotechnology Inc., Beijing 100176, China; yj.liu@bj-klws.com (Y.L.); hy.yu@bj-klws.com (H.Y.)

**Keywords:** human papillomavirus, nonavalent, *Escherichia coli*, phase 1 clinical trial, safety, immunogenicity

## Abstract

Background: Prophylactic human papillomavirus (HPV) vaccination substantially alleviates cervical cancer burden. This study aimed to evaluate the safety, tolerability, and immunogenicity of an *Escherichia coli*-expressed recombinant nonavalent HPV vaccine. Methods: A dose-escalating phase 1 clinical trial was conducted in Sheyang County, Jiangsu Province, China. Each participant received either the test vaccine or the control vaccine (Gardasil 9) following a 0/2/6-month schedule. Adverse reactions (ARs) within 7 days after vaccination, adverse events (AEs) within 30 days, and serious adverse events (SAEs) throughout the study were recorded. Blood parameters were measured before and 3 days after each dose. Serum immunoglobulin G (IgG) and neutralizing antibodies (nAbs) against nine HPV types were analyzed at months 0, 3, and 7. Results: A total of 160 women aged 18–45 years were enrolled, and 155 participants completed the full vaccination regimen. Within 7 days following vaccination, the incidence of ARs ranged from 56.67% to 90.00%, with the low-dose group showing a significantly higher rate than the control group (*p* = 0.004). Most AEs were mild or moderate, and no vaccine-related SAEs occurred. No significant differences were observed among the four groups regarding the incidence of abnormal laboratory findings. Seroconversion rates for nAbs and IgG against nine HPV types exceeded 97.92% following three doses. High levels of nAbs and IgG were observed at months 3 and 7, with geometric mean titers (GMTs) showing further increases by month 7. Conclusions: This new recombinant nonavalent HPV vaccine exhibits good tolerability and strong immunogenicity among women aged 18–45 years, supporting further efficacy studies in larger populations.

## 1. Introduction

Human papillomavirus (HPV) is the leading etiological agent of cervical cancer, which is recognized as the fourth most prevalent malignant cancer among women globally [1]. In 2022, an estimated 662,301 new cervical cancer cases and 348,874 related deaths were reported worldwide [2]. In China alone, approximately 150,000 cervical cancer cases emerged, and 55,000 people died from this cancer in 2022 [3]. Nearly all cervical cancer cases, along with a large proportion of anogenital and oropharyngeal cancers, as well as genital warts, are caused by persistent HPV infection [4]. Over 70% of cervical cancer cases are attributable to the high-risk HPV types 16 and 18 [5], while about 90% of genital warts cases are caused by low-risk HPV types 6 and 11 [6].

Prophylactic HPV vaccination plays a crucial role in mitigating the global disease burden caused by HPV [7]. In 2020, the World Health Organization (WHO) officially introduced the Cervical Cancer Elimination Strategy, aiming to make cervical cancer no longer a public health concern by the year 2030. A key objective of this strategy is to ensure that 90% of girls have completed the HPV vaccination series by the age of 15 [8]. Currently, six licensed HPV vaccines have been approved, all of which demonstrate favorable safety profiles and effectiveness in preventing HPV infection. These include three bivalent vaccines targeting HPV types 16 and 18, two quadrivalent vaccines targeting HPV6, 11, 16, and 18, and a nonavalent vaccine covering HPV6, 11, 16, 18, 31, 33, 45, 52, and 58. Gardasil 9, the nonavalent HPV vaccine developed by Merck Sharp & Dohme Corp., Rahway, NJ, USA, incorporates five extra high-risk HPV types (31, 33, 45, 52, and 58), thereby expanding cervical cancer prevention coverage from approximately 70% to 90% [9]. However, Gardasil 9 remains largely inaccessible in low-resource settings due to limited production and high cost [10]. This presents a major obstacle for low- and middle-income countries, which bear 88.1% of the global cervical cancer burden [9].

Of the six licensed HPV vaccines, a bivalent HPV 16/18 vaccine (Cecolin; Xiamen Innovax, Xiamen, China) produced by *Escherichia coli* (*E. coli*) expression platform was launched in China in 2019 and obtained WHO prequalification in 2021 [11]. Its affordability and scalable production make it a promising solution to address vaccine shortages and expand coverage [12,13]. Our candidate recombinant nonavalent HPV vaccine, also produced using an *E. coli* expression system similar to Cecolin, may offer an expanded option for preventing HPV-related diseases. Here, we report the safety and immunogenicity results of it from a phase 1 clinical trial conducted in healthy Chinese women aged 18–45 years.

## 2. Materials and Methods

### 2.1. Study Design and Participants

This single-center, dose-escalating phase 1 clinical trial was conducted to evaluate the safety, tolerability, and immunogenicity of a recombinant nonavalent HPV vaccine (*E. coli*) in healthy Chinese women from September 2019 to May 2020. The trial received approval from the Independent Ethics Committee of the Jiangsu Provincial Center for Disease Control and Prevention (JSJK2019-A003-01) and was performed in accordance with the principles of the Declaration of Helsinki and Good Clinical Practice guidelines. The study was registered at ClinicalTrials.gov (NCT05680454).

**Figure 1:**
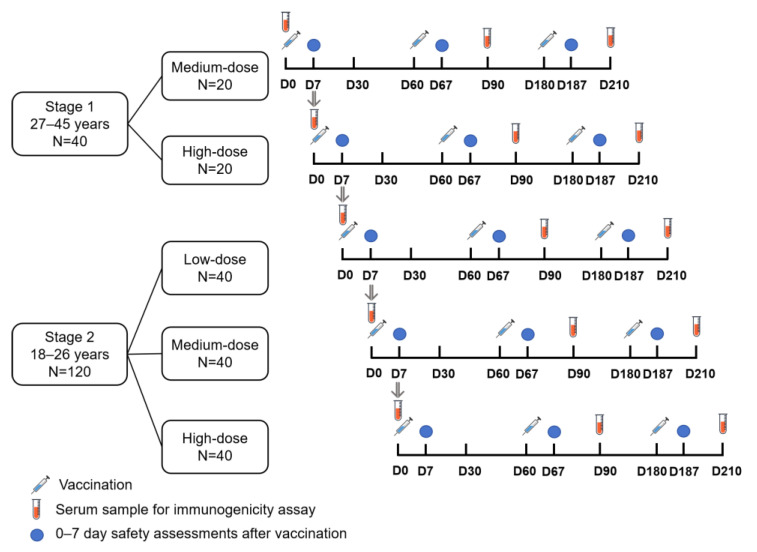
Overview of study design.

An overview of the study design is shown in Figure 1. The study was conducted in two stages, following a sequential and dose-escalating approach. Based on a 7-day safety follow-up, dose escalation could be paused if any safety risks were observed. The first stage was an open-label trial. The medium-dose group enrolled 20 participants in two steps (5 and 15 participants) with a 3-day interval. The high-dose group followed the same enrollment process after confirming safety. In total, 40 women aged 27–45 years received either medium-dose or high-dose test vaccine. The second stage was a randomized, blinded, and positive-controlled trial in which 120 women aged 18–26 years were divided into low-dose, medium-dose, and high-dose groups (40 per group). Participants in each dose group received either the test or control vaccine (Gardasil 9) in a 3:1 ratio. Enrollment for the low-dose group was carried out in four steps (5, 10, 10, and 15 participants, respectively) with a 3-day interval between each step. Subsequently, the same enrollment process was adopted for the medium-dose and high-dose groups. Each participant was under follow-up for approximately 7 months.

Healthy women aged 18–45 years residing in Sheyang County, Yancheng, Jiangsu, China, were recruited for the study. Written informed consent was obtained from all enrolled individuals prior to the initiation of any study procedures. Eligibility criteria included an age range of 18 to 45 years, normal axillary temperature (≤37.0 °C), a negative result on a urine pregnancy test, and either no childbearing potential or childbearing potential with a requirement to abstain from sexual activity. Women who were pregnant or breastfeeding, had received any HPV vaccine previously, had a history of severe allergic reactions with vaccines, or had an autoimmune disease or immunodeficiency were excluded. A complete list of the inclusion and exclusion criteria is provided in the Supplementary Materials.

### 2.2. Randomization and Masking

The first stage was an open-label trial. The second stage used stratified block randomization to ensure balance across different dose groups (low-dose, medium-dose, and high-dose groups). Eligible participants were stratified by dose level, and within each stratum, randomization lists were generated within each stratum using SAS version 9.4 software. In the second stage, participants were randomly assigned in a 3:1 ratio to receive three doses of either the test vaccine or the positive control vaccine. Each participant was assigned a unique vaccine code sequentially, based on their enrollment order. To maintain the blinding of the study, both the test and control vaccines were indistinguishable in terms of outer packaging, labeling, and volume. Due to differences in the inner packaging, the personnel responsible for vaccine retrieval and administration were aware of the subject allocation, but they were not permitted to participate in any other aspects of the study, including follow-up. The allocation of participants to study groups and vaccine codes remained blinded to all participants, investigators, and study center personnel.

### 2.3. Procedures

The recombinant nonavalent HPV vaccine (types 6/11/16/18/31/33/45/52/58), derived from *E. coli*, was manufactured by Beijing Health Guard Biotechnology Inc., Beijing, China. The HPV L1 proteins for each type were produced in *E. coli* and purified to obtain high-purity HPV L1 pentamer proteins. Partial truncations were made at both the N- and C-termini of the L1 protein to improve the solubility of the L1 pentamer. This modification enhanced the expression of L1 pentamer proteins in the bacterial expression system and prevented degradation of the terminal amino acids that could affect the integrity and stability of the virus-like particles (VLPs). Each 0.5 mL dose of the vaccine contains a specific amount of HPV L1 VLPs, with the low-dose group, medium-dose group, and high-dose group containing a total of 220 µg, 270 µg, and 360 µg, respectively. The control vaccine used in this study was Gardasil 9 (Merck Sharp & Dohme Corp., Rahway, NJ, USA), which was first marketed in China in 2018. Both vaccines were administered by intramuscular injection into the deltoid muscle of the upper arm at 0, 2, and 6 months.

Following each vaccination, participants were monitored on site for 30 min to detect any immediate adverse events (AEs). They were also instructed on the use of diary cards to document solicited local and systemic adverse reactions (ARs) for 7 days, as well as any AEs occurring within 30 days following each vaccination. Serious adverse events (SAEs) were recorded throughout the entire study duration. Blood samples were collected prior to and on day 3 after each vaccination for routine hematological and biochemical analyses. Laboratory indices included two routine blood tests, white blood cell count (WBC) and hemoglobin (HGB), three serum biochemical tests, creatinine (CREA), alanine aminotransferase (ALT), and aspartate aminotransferase (AST), and two blood coagulation function tests, prothrombin time (PT) and activated partial thromboplastin time (APTT). The severity grading of both AEs and abnormal lab findings was carried out by investigators according to the Guidelines for Adverse Event Classification Standards for Clinical Trials of Preventive Vaccines (2019) issued by the National Medical Products Administration (NMPA).

Serum samples were collected from all participants on day 0, month 3 (30 days after the second vaccination), and month 7 (30 days after the third vaccination) to evaluate the levels of HPV 6/11/16/18/31/33/45/52/58 type-specific immunoglobulin G (IgG) and neutralizing antibodies (nAbs). These samples were also tested for anti-glutathione-S-transferase (GST) and 3C protease-related antibodies. Detection of IgG antibodies was performed using an enzyme-linked immunosorbent assay (ELISA) based on HPV VLPs produced in *E. coli*, while neutralizing antibodies were assessed using a pseudovirion-based neutralization assay (PBNA). The IgG and neutralizing antibody tests in this study were carried out at the China National Institute for Food and Drug Control (NIFDC), and the anti-GST and 3C protease-related antibody tests were conducted at Beijing Health Guard Biotechnology Inc.

### 2.4. Outcomes

The primary outcome was the incidence of ARs within 7 days post each vaccination. Secondary safety outcomes included the incidence of AEs within 30 days, the percentage of abnormal blood routine indexes, blood biochemical indexes, and coagulation time within 3 days after each dose, as well as the overall rate of SAEs recorded throughout the study. Secondary immunogenicity outcomes included the seroconversion rates and levels of HPV 6/11/16/18/31/33/45/52/58 type-specific nAbs and IgG antibodies at months 3 and 7 post-vaccination. Subgroup analyses were conducted on participants with negative pre-immunization antibodies against nine HPV subtypes, evaluating their seroconversion rates and antibody responses at months 3 and 7 post-vaccination. Exploratory outcomes included the levels of anti-GST and 3C protease-related antibodies at baseline and at months 3 and 7 post-vaccination.

### 2.5. Statistical Analysis

The sample size was determined in accordance with the Technical Guidelines for Vaccine Clinical Trials issued by the NMPA, which recommends a sample size of 20 to 30 participants for Phase 1 clinical trials.

The safety set (SS) included all individuals who received at least one vaccine dose. The full analysis set (FAS), closely aligned with the intention-to-treat principle, comprised participants who received at least one dose and provided at least one serum sample. Immunogenicity assessments were performed using the per-protocol set (PPS), consisting of participants who satisfied all eligibility criteria, completed the three-dose vaccination schedule, and submitted blood samples as per the study protocol. Seroconversion was defined as either a change from seronegative at baseline to seropositive after vaccination, or at least a four-fold increase in antibody titers over baseline. The geometric mean increase (GMI) refers to the geometric mean of fold increase in the antibody titers.

All AEs were summarized by dose group as frequencies and percentages. Differences in AE incidence rates across groups were calculated using Pearson’s Chi-squared test or Fisher’s exact test. The geometric mean titer (GMT) and GMI for both IgG and nAbs, along with their 95% confidence intervals (CIs), were calculated based on Student’s t distribution of the log-transformed antibody titers. The Wilcoxon rank-sum test was used to analyze data that did not follow a normal distribution. Seroconversion rates and antibody titers were compared across the four groups using Pearson’s Chi-squared test and an ANOVA, respectively. For multiple comparisons, the significance level was adjusted using the Bonferroni correction, with an adjusted α value of 0.008. Differences between groups were considered statistically significant if *p* < 0.008 for multiple comparisons. For all other analyses, *p* < 0.05 was considered statistically significant. Data were analyzed using SAS version 9.4 (SAS Institute Inc., Cary, NC, USA), and GraphPad Prism 9.5 (GraphPad Software, Inc., San Diego, CA, USA) was used for graphing.

## 3. Results

### 3.1. Baseline Demographic Characteristics

**Figure 2:**
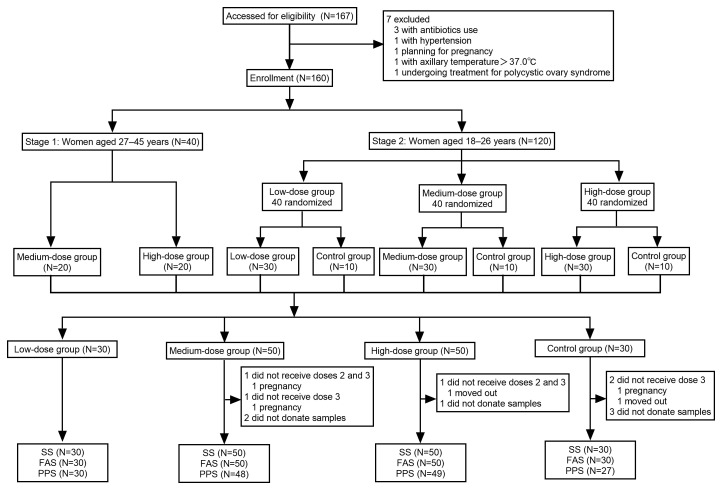
Trial profile. The dose-escalation phase 1 study was carried out in two stages. Recruitment for Stage 2 commenced only after confirming the absence of vaccine-related serious adverse events within 7 days following the first dose administered in Stage 1. All the participants received three doses of the allocated vaccine according to the protocol. SS: safety analysis set; FAS: full analysis set; PPS: per-protocol set.

From 1 September 2019 to 10 May 2020, a total of 167 women aged 18–45 years were screened for eligibility, with 160 healthy women ultimately included in the study. In Stage 1, 40 women aged 27–45 years were included, while in Stage 2, 120 women aged 18–26 years were recruited (Figure 2). Participants in Stage 2 were only included after confirming the absence of vaccine-related SAEs within 7 days following the first dose administered to the participants in Stage 1. Among the enrolled participants, five did not complete the full vaccination schedule, and six failed to provide serum samples at all three scheduled time points (months 0, 3, and 7). The low-dose and control groups each had 30 participants, while the medium-dose and high-dose groups each had 50 participants. Baseline demographic characteristics were comparable across all groups (Table 1).

### 3.2. Safety and Tolerability

All 160 participants enrolled in the study received at least one dose of the vaccine and were included in the safety analysis set. Within 7 days following each vaccination, a total of 338 ARs were recorded (Table 2). The overall incidence of ARs was 90.00% in the low-dose group, 74.00% in the medium-dose group, 74.00% in the high-dose group, and 56.67% in the control group. A significant difference was observed between the low-dose and control groups (*p* = 0.004). Similarly, the rate of solicited ARs was also higher in the low-dose group compared to the control group (90.00% vs. 56.67%, *p* = 0.004). The incidences of unsolicited AEs remained comparable across the groups, with rates of 6.67%, 8.00%, 8.00%, and 6.67%, respectively. Most reported ARs were mild or moderate. Two participants reported Grade 3 ARs, one being induration and the other being fever, both of which occurred in the high-dose group (Table S3). No serious adverse reaction occurred during the study.

**Figure 3:**
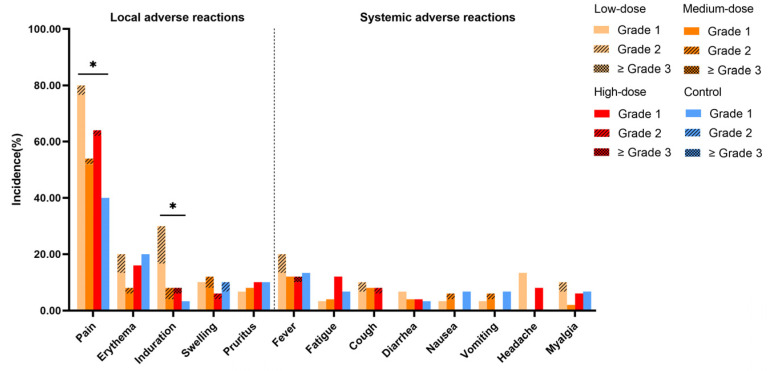
The incidences of solicited adverse reactions within 7 days after each vaccination. The multiple comparisons were adjusted using the Bonferroni method, with αadjusted = α/6 = 0.008. Differences were considered statistically significant if *p* < 0.008. * The significance is at the *p* < 0.008 level.

The most frequently reported local AR within 7 days post-vaccination was pain at the injection site, occurring in 59.38% of participants. A statistically significant difference in incidence was found between the low-dose and control groups (80.00% vs. 40.00%, *p* = 0.006) (Figure 3). Erythema was also commonly reported, with incidences of 20.00%, 8.00%, 16.00%, and 20.00% in the low-dose, medium-dose, high-dose, and control groups, respectively. Induration was reported in 30.00%, 8.00%, 8.00%, and 3.33% of participants in the four groups, with the low-dose group showing a significantly higher incidence than the control group (*p* = 0.006). The most common systemic ARs were fatigue (13.75%), cough (7.50%), and headache (6.88%), with no significant differences observed among the groups.

The overall incidence of AEs within 30 days post-vaccination was as follows: 90.00% in the low-dose group, 80.00% in the medium-dose group, 82.00% in the high-dose group, and 66.67% in the control group (Table 3). The incidence of total ARs ranged from 56.67% to 90.00%, with a significant difference found between the low-dose and control groups (*p* = 0.004). Notably, the rate of solicited AEs was significantly higher in the low-dose group compared to the control group (90.00% vs. 56.67%, *p* = 0.004). Most reported AEs were mild or moderate. As described before, two Grade 3 AEs occurred within 7 days post-vaccination in the high-dose group (Table S3). During the entire study period, one serious adverse event (SAE) occurred more than 30 days after vaccination. The investigators ultimately determined that it was unrelated to the vaccination.

When stratified by age, a significant difference was observed in the incidence of total AEs within 30 days post-vaccination in women aged 27–45 years, with the medium-dose group showing a lower incidence compared to the high-dose group (65.00% vs. 95.00%, *p* = 0.048) (Table S1). For women aged 18–26 years, another significant difference was found in the incidence of total ARs between the low-dose and control groups (90.00% vs. 56.67%, *p* = 0.004) (Table S2).

### 3.3. Laboratory Parameters

A total of seven hematological parameters were analyzed to assess changes before and 3 days after each dose of vaccination. Table 4 summarizes the post-vaccination abnormalities, defined as cases where the test result was “normal” prior to vaccination but became “abnormal” afterward. The incidence of abnormalities in various indicators following vaccination was generally low. Among these, the highest incidence was observed in abnormal WBC and CREA levels after the third dose, with both showing an incidence of 5.19%. No significant differences were observed among the four groups regarding the incidence of abnormal laboratory findings. The majority of post-vaccination abnormalities were classified as mild or moderate and were deemed clinically insignificant by clinicians.

### 3.4. Immunogenicity

The seroconversion rates for nAbs and IgG against HPV types 6/11/16/18/31/33/45/52/58 at month 3 (one month after the second dose) in the PPS cohorts are presented in Table S4. In the low-dose group, all participants achieved seroconversion for both nAbs and IgG against all nine HPV types. Within the medium-dose group, one participant did not seroconvert for nAbs of HPV 45 (seroconversion rate: 97.92%), and two did not seroconvert for nAbs of HPV 58 (seroconversion rate: 95.83%). Similarly, in the high-dose group, one participant failed to seroconvert for nAbs against HPV 45 and HPV 58, with seroconversion rates of 97.96% for each. In the control group, one participant did not achieve seroconversion for nAbs against HPV 45 (seroconversion rate: 96.30%), three failed to seroconvert for nAbs of HPV 58 (seroconversion rate: 88.89%), and one failed to seroconvert for IgG against HPV 6 and HPV 11 (seroconversion rate: 96.30% for both).

At month 7, all participants in the PPS cohort achieved seroconversion for IgG against all nine HPV types, while one participant in the medium-dose group and another in the control group failed to seroconvert for nAbs against HPV 45 (seroconversion rates: 97.92% and 96.30%, respectively) (Table S5). Additionally, one participant in the high-dose group did not achieve seroconversion for nAbs against HPV 31 and HPV 58 (seroconversion rate: 97.96% for both).

**Figure 4:**
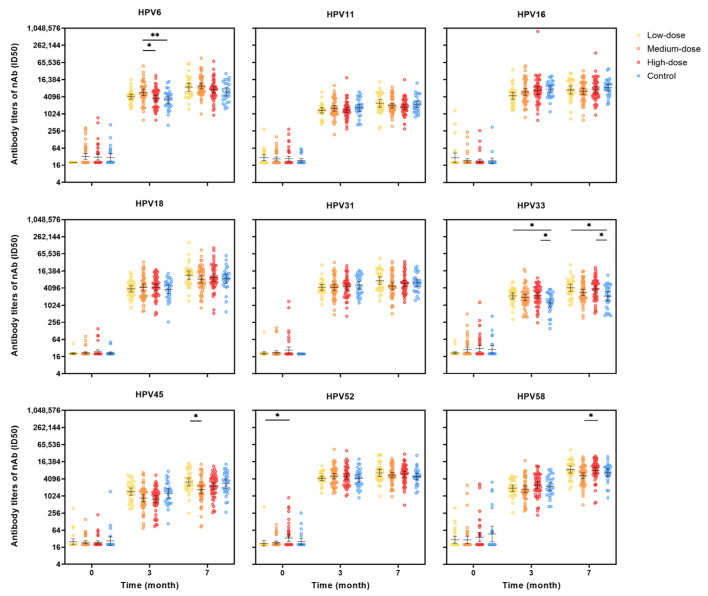
Neutralizing antibody titers at months 0, 3, 7 for HPV types 6/11/16/18/31/33/45/52/58 in the PPS cohort. PPS: per-protocol set, which included participants who received all three vaccine doses and donated serum samples at months 0, 3, and 7, and had no major deviation of the protocol; The antibody level of the seronegative sample was artificially set as half of the cutoff value; The black lines indicate the GMTs and 95% CI; CI: confidence interval; GMTs: geometric mean titers; nAb: neutralizing antibody. The multiple comparisons were adjusted using the Bonferroni method, with αadjusted = α/6 = 0.008. Differences were considered statistically significant if *p* < 0.008. * The significance is at the *p* < 0.008 level. ** The significance is at the *p* < 0.001 level.

The GMTs of nAbs for all nine HPV types showed substantial increases one month after the administration of two vaccine doses. A further increase in nAb titers was observed one month after the third dose, which was administered at month 6 (Figure 4). Notably, no clear dose-dependent escalation in antibody responses was observed among the three dose groups. Overall, the GMTs of antibodies were comparable across the different dose groups and the control group, except for the GMT of HPV 6 nAbs, which was significantly higher in the medium-dose group compared to the other groups. The GMT of HPV 33 nAbs was significantly higher in the high-dose group, while the GMT of HPV 45 nAbs was higher in the low-dose group compared to the other groups.

**Figure 5:**
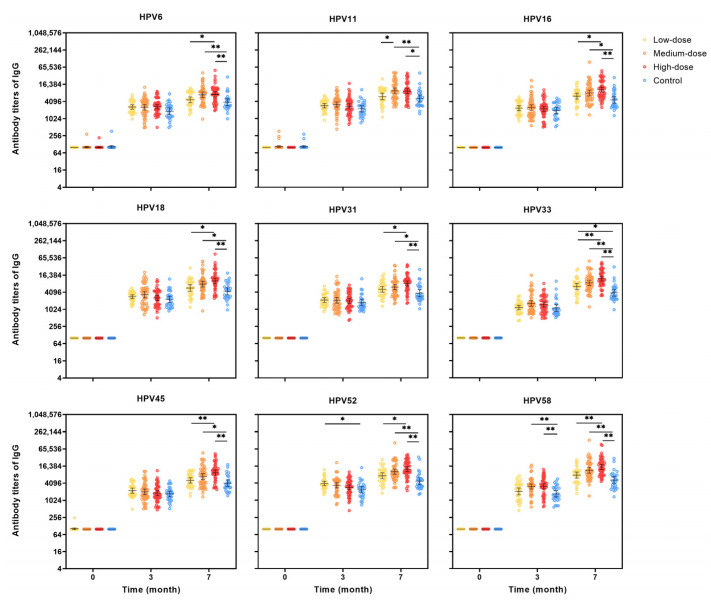
IgG antibody titers at months 0, 3, 7 for HPV types 6/11/16/18/31/33/45/52/58 in the PPS cohort. PPS: per-protocol set, which included participants who received all three vaccine doses and donated serum samples at months 0, 3, and 7, and had no major deviation of the protocol; The antibody level of the seronegative sample was artificially set as half of the cutoff value; The black lines indicate the GMTs and 95% CI; CI: confidence interval; GMTs: geometric mean titers. The multiple comparisons were adjusted using the Bonferroni method, with αadjusted = α/6 = 0.008. Differences were considered statistically significant if *p* < 0.008. * The significance is at the *p* < 0.008 level. ** The significance is at the *p* < 0.001 level.

Similar trends were observed in the IgG antibody data (Figure 5). Significant differences in GMTs were noted among the groups for all nine HPV types. Specifically, the GMT of HPV 11 IgG was significantly higher in the medium-dose group compared to the others, while the low-dose group showed elevated IgG GMTs for the remaining HPV types.

All participants who were seronegative for nine HPV types at baseline successfully seroconverted for both nAbs and IgG against all HPV types at month 7. The GMI and GMTs for both nAbs and IgG at months 3 and 7 in these participants are presented in Tables S6 and S7, and Figures S1 and S2.

The exploratory analysis revealed that the levels of antibodies against GST and 3C protease at baseline and at months 3 and 7 post-vaccination were negative across all groups, with no statistically significant differences observed between the groups.

## 4. Discussion

This phase 1 clinical trial preliminarily indicated that the recombinant nonavalent HPV vaccine (*E. coli*) is safe and well tolerated in healthy women aged 18–45 years. Our findings show that the incidence of total ARs within 7 days after vaccination ranged from 56.67% to 90.00%, with a higher incidence in the low-dose group compared to the control group. Most reported AEs were mild or moderate, and no vaccine-related SAEs were reported. Laboratory findings further corroborated the favorable safety profile of the test vaccine. No significant differences were found in the incidence of abnormal laboratory parameters within 3 days of each dose across all four groups. Additionally, the exploratory analysis revealed that antibody levels against GST and 3C protease were negative at baseline and at months 3 and 7 post-vaccination across all groups, further confirming the safety of the trial vaccine.

Within 30 days following vaccination, the reported incidence of AEs was 90.00% in the low-dose group, 80.00% in the medium-dose group, and 82.00% in the high-dose group, which is comparable to the 83.30% observed with the *E. coli*-expressed 9vHPV vaccine (Cecolin9, Xiamen Innovax, Xiamen, China) [14]. Previous studies on the 9-valent HPV vaccine showed AR incidence rates of 60.00–70.00% in adults, with injection-site pain reported in 60.00% of participants [15]. In comparison to Gardasil 9, the candidate vaccine demonstrated a higher incidence of ARs within 7 days post-vaccination, which may be attributed to differences between the adjuvants and the production systems used [16]. The recombinant nonavalent HPV vaccine uses *E. coli*-derived VLPs (a prokaryotic system), whereas Gardasil 9 employs a yeast-derived system (eukaryotic). Prokaryotic systems like *E. coli* may introduce higher levels of endotoxins and bacterial proteins, which can lead to stronger local inflammatory responses, thus increasing the occurrence of ARs [17].

Furthermore, the low-dose group exhibited a significantly higher incidence of solicited reactions than the control group (90.00% vs. 56.67%, *p* = 0.004), such as injection-site pain (80.00% vs. 40.00%, *p* = 0.002) and induration (30.00% vs. 3.33%, *p* = 0.006). This elevated reactogenicity may be attributed to a stronger immune response triggered by the lower antigen dose, particularly at the injection site. These findings indicate that with similarly good immunogenicity, the medium-dose and high-dose options may be more suitable for further evaluation.

Robust immunogenicity was observed after three doses of the vaccine. The seroconversion rates for nAbs and IgG against all nine HPV types were over 97.92% at 7 months post-vaccination, which is similar to the findings from the phase 3 study of Gardasil 9 (99.60–100.00%) [18]. The GMTs for all nine HPV types showed a marked rise as early as one month after the second dose (month 3), supporting the WHO’s 2022 recommendation for two-dose schedules of HPV vaccine, especially in resource-limited settings [7]. Our findings suggest the potential for a two-dose nonavalent HPV vaccine, laying the groundwork for future trials assessing optimized two-dose regimens for women [10]. A further increase in nAb and IgG titers was observed one month after the third dose, which was administered at month 6. The robust immunogenic responses observed in this trial are consistent with results from early-phase studies of other *E. coli*-derived HPV vaccines. For instance, a phase 1 study evaluating an *E. coli*-based bivalent HPV vaccine reported similarly high seroconversion rates and GMTs for HPV 6/11 [19]. Similarly, a phase 2 trial of Cecolin9, also manufactured using *E. coli*, demonstrated a comparable rise in GMTs for all nine HPV types one month after both the second and third doses [10].

Given that HPV infects through the mucosal epithelium, mucosal immunity plays an important role in protection, with secretory immunoglobulin A (sIgA) serving as a key neutralizing antibody at mucosal surfaces. Previous studies have shown that intramuscularly administered HPV vaccines, such as Gardasil, can induce detectable levels of mucosal IgA [20]. As the candidate vaccine is produced using an *E. coli* expression system, its ability to induce mucosal immune responses, particularly sIgA at the cervicovaginal site, should be further investigated to provide a more comprehensive understanding of its protective potential.

The nAb levels against the nine HPV types across three dose groups were comparable to those in the control group. However, IgG GMTs varied significantly among the groups for all nine HPV types. Specifically, the medium-dose group exhibited higher GMTs for HPV 11 IgG, whereas the low-dose group showed significantly higher IgG levels for the remaining HPV types compared to the other groups, suggesting no dose-dependent immune responses across the dose groups. This may be attributed to the early saturation of immune responses, where even lower doses of the vaccine are sufficient to induce a strong immune reaction [21].

This study has several limitations. First, the study did not evaluate the immunization schedules of a single dose or two doses with a 6-month interval in parallel. Since the WHO recently recommended a two-dose regimen for adults, further research on this schedule is needed to provide additional evidence for policy-making. Second, in the analysis of the primary outcome, a per-protocol analysis was adopted, excluding those who did not complete the full vaccination and missed serum antibody results, which could potentially introduce selection bias. Additionally, the trial only includes women aged 18–45 years, which may not fully represent the broader population who could benefit from the vaccine. Other age groups, particularly adolescents aged 9–14 years (the target population for HPV vaccination), should be included in future studies.

## 5. Conclusions

In conclusion, this phase 1 clinical study demonstrated that the new recombinant nonavalent HPV vaccine (*Escherichia coli*) exhibits good tolerability and strong immunogenicity in healthy Chinese women aged 18–45 years. These findings indicate that further investigation is warranted in broader age groups and larger cohorts to assess its safety, immunogenicity, and protective efficacy.

## Figures and Tables

**Table 1:** Baseline demographic characteristics of the participants.

Characteristics	Low-Dose (N = 30)	Medium-Dose (N = 50)	High-Dose (N = 50)	Control (N = 30)	Total (N = 160)	*p* Value
Age (years)						
Mean (SD)	22.60 (2.18)	28.90 (8.28)	28.52 (8.10)	22.73 (2.41)	26.44 (7.20)	<0.001
Median	23.00	25.50	24.50	23.00	24.00	
Min, Max	18.00, 26.00	18.00, 45.00	19.00, 45.00	18.00, 26.00	18.00, 45.00	
Height (cm)						
Mean (SD)	160.07 (5.12)	160.90 (5.46)	161.14 (5.67)	161.63 (7.65)	160.96 (5.90)	0.748
Median	160.50	161.00	161.00	162.00	161.00	
Min, Max	151.00, 171.00	151.00, 172.00	146.00, 173.00	143.00, 181.00	143.00, 181.00	
Weight (kg)						
Mean (SD)	57.97 (9.61)	59.54 (12.32)	60.54 (10.39)	58.57 (11.20)	59.38 (10.99)	0.678
Median	55.50	58.50	58.50	55.00	58.00	
Min, Max	44.00, 80.00	41.00, 115.00	45.00, 92.00	41.00, 97.00	41.00, 115.00	
BMI (kg/m^2^)						
Mean (SD)	22.63 (3.62)	22.94 (4.23)	23.34 (3.99)	22.33 (3.20)	22.89 (3.85)	0.693
Median	21.77	22.74	22.81	21.75	22.40	
Min, Max	16.94, 31.64	15.06, 40.75	15.57, 35.94	16.63, 29.61	15.06, 40.75	

SD: standard deviation. N: the number of people in each dataset. Age = (date of informed consent − date of birth)/365.25. *p* values were calculated using Pearson’s Chi-squared test or Fisher’s exact test.

**Table 2:** Adverse reactions within 7 days after each vaccination.

	Low-Dose (N = 30)		Medium-Dose (N = 50)		High-Dose (N = 50)		Control (N = 30)		Total (N = 160)		*p* Value
	Events	Participants (%)	Events	Participants (%)	Events	Participants (%)	Events	Participants (%)	Events	Participants (%)	
Total adverse reactions ^a^	92	27 (90.00)	86	37 (74.00)	108	37 (74.00)	52	17 (56.67)	338	118 (73.75)	0.035 ^b^
Solicited adverse reactions	90	27 (90.00)	82	35 (70.00)	104	36 (72.00)	49	17 (56.67)	325	115 (71.88)	0.039 ^b^
Local solicited adverse reactions	65	25 (83.33)	59	30 (60.00)	72	34 (68.00)	36	16 (53.33)	232	105 (65.63)	0.072
Systemic solicited adverse reactions	25	13 (43.33)	23	15 (30.00)	32	16 (32.00)	13	7 (23.33)	93	51 (31.88)	0.407
Unsolicited adverse reactions	2	2 (6.67)	4	4 (8.00)	4	4 (8.00)	3	2 (6.67)	13	12 (7.50)	>0.999
Adverse reactions ≥ grade 3	0	0	0	0	2	2 (4.00)	0	0	2	2 (1.25)	0.332

^a^ Any vaccine-related adverse events. ^b^ The low-dose group showed a higher incidence than did the control group (*p* = 0.004). Except for the occurrences of ARs, each subject in each trial group was counted only once per row. *p* values were calculated using Pearson’s Chi-squared test or Fisher’s exact test. The multiple comparisons were adjusted using the Bonferroni method, with αadjusted = α/6 = 0.008. Differences were considered statistically significant if *p* < 0.008.

**Table 3:** Adverse events within 30 days after each vaccination.

	Low-Dose (N = 30)		Medium-Dose (N = 50)		High-Dose (N = 50)		Control (N = 30)		Total (N = 160)		*p* Value
	Events	Participants (%)	Events	Participants (%)	Events	Participants (%)	Events	Participants (%)	Events	Participants (%)	
Total adverse events	112	27 (90.00)	106	40 (80.00)	131	41 (82.00)	63	20 (66.67)	412	128 (80.00)	0.149
Adverse reactions ^a^	93	27 (90.00)	86	37 (74.00)	111	37 (74.00)	52	17 (56.67)	342	118 (73.75)	0.035 ^b^
Solicited adverse reactions	90	27 (90.00)	82	35 (70.00)	104	36 (72.00)	49	17 (56.67)	325	115 (71.88)	0.039 ^b^
Local solicited adverse reactions	65	25 (83.33)	59	30 (60.00)	72	34 (68.00)	36	16 (53.33)	232	105 (65.63)	0.072
Systemic solicited adverse reactions	25	13 (43.33)	23	15 (30.00)	32	16 (32.00)	13	7 (23.33)	93	51 (31.88)	0.407
Unsolicited adverse reactions	3	3 (10.00)	4	4 (8.00)	7	5 (10.00)	3	2 (6.67)	17	14 (8.75)	0.981
Solicited adverse events	90	27 (90.00)	82	35 (70.00)	109	36 (72.00)	49	17 (56.67)	330	115 (71.88)	0.039 ^b^
Unsolicited adverse events	22	12 (40.00)	24	14 (28.00)	22	14 (28.00)	14	9 (30.00)	82	49 (30.63)	0.666
Adverse events ≥ grade 3	0	0	0	0	2	2 (4.00)	0	0	2	2 (1.25)	0.332
Serious adverse events	0	0	0	0	0	0	0	0	0	0	-
Discontinuation due to adverse events	0	0	0	0	0	0	0	0	0	0	-

^a^ Any vaccine-related adverse events. ^b^ The low-dose group showed a higher incidence than did the control group (*p* = 0.004). Except for the occurrences of AEs, each subject in each trial group was counted only once per row. *p* values were calculated using Pearson’s Chi-squared test or Fisher’s exact test. The multiple comparisons were adjusted using the Bonferroni method, with αadjusted = α/6 = 0.008. Differences were considered statistically significant if *p* < 0.008.

**Table 4:** Summary of abnormal laboratory parameters after each dose of vaccination.

	Indices	Low-Dose (N = 30)	Medium-Dose (N = 50)	High-Dose (N = 50)	Control (N = 30)	Total (N = 160)	*p* Value
Routine blood indices	WBC						
	1st vaccination	0	3 (6.00)	2 (4.00)	1 (3.33)	6 (3.75)	0.702
	2nd vaccination	0	0	1 (2.04)	0	1 (0.63)	>0.999
	3rd vaccination	3 (10.00)	2 (4.17)	3 (6.12)	0	8 (5.19)	0.421
	HGB						
	1st vaccination	0	0	0	0	0	-
	2nd vaccination	0	1 (2.04)	2 (4.08)	0	3 (1.90)	0.777
	3rd vaccination	0	1 (2.08)	0	0	1 (0.65)	0.682
Serum biochemical indices	ALT						
	1st vaccination	1 (3.33)	0	1 (2.00)	0	2 (1.25)	0.803
	2nd vaccination	1 (3.33)	1 (2.04)	1 (2.04)	0	3 (1.90)	>0.999
	3rd vaccination	1 (3.33)	2 (4.17)	0	0	3 (1.95)	0.398
	AST						
	1st vaccination	0	0	0	0	0	-
	2nd vaccination	0	1 (2.04)	1 (2.04)	0	2 (1.27)	>0.999
	3rd vaccination	1 (3.33)	1 (2.08)	0	0	2 (1.30)	0.676
	CREA						
	1st vaccination	1 (3.33)	0	1 (2.00)	0	2 (1.25)	0.803
	2nd vaccination	0	0	4 (8.16)	0	4 (2.53)	0.068
	3rd vaccination	2 (6.67)	3 (6.25)	1 (2.04)	2 (7.41)	8 (5.19)	0.667
Blood coagulation function indices	APTT						
	1st vaccination	0	1 (2.00)	1 (2.00)	0	2 (1.25)	>0.999
	2nd vaccination	0	2 (4.08)	2 (4.08)	2 (6.67)	6 (3.80)	0.568
	3rd vaccination	0	2 (4.17)	0	1 (3.70)	3 (1.95)	0.288
	PT						
	1st vaccination	1 (3.33)	0	0	1 (3.33)	2 (1.25)	0.139
	2nd vaccination	0	2 (4.08)	0	0	2 (1.27)	0.332
	3rd vaccination	1 (3.33)	1 (2.08)	1 (2.04)	1 (3.70)	4 (2.60)	>0.999

WBC: white blood cell count; HGB: hemoglobin; ALT: alanine aminotransferase; AST: aspartate aminotransferase; CREA: creatinine; APTT: activated partial thromboplastin time; PT: prothrombin time. Post-vaccination abnormality: The test result before vaccination was “normal”, while the test result after vaccination was “abnormal”. Data for the 1st, 2nd, and 3rd dose analyses were based on SS1, SS2, and SS3, respectively. Data are n (%), unless stated otherwise. *p* values were calculated using Pearson’s Chi-squared test or Fisher’s exact test.

## Data Availability

The data presented in this study are available upon request from the corresponding author.

## Supplementary Materials

The following supporting information can be downloaded at https://www.mdpi.com/article/10.3390/vaccines13050511/s1, Table S1: Adverse events within 30 days after each vaccination in women aged 27–45 years. Table S2: Adverse events within 30 days after each vaccination in women aged 18–26 years. Table S3: List of adverse events grade ≥ 3 that occurred within 30 days after vaccination. Table S4: Seroconversion rates of HPV type-specific neutralizing and IgG antibodies at month 3 in the PPS cohort. Table S5: Seroconversion rates of HPV type-specific neutralizing and IgG antibodies at month 7 in the PPS cohort. Table S6: Geometric mean increases of HPV type-specific neutralizing and IgG antibodies at month 3 in the participants who were seronegative for HPV types at baseline. Table S7: Geometric mean increases of HPV type-specific neutralizing and IgG antibodies at month 7 in the participants who were seronegative for HPV types at baseline. Figure S1: Neutralizing antibody titers at months 3 and 7 for HPV types 6/11/16/18/31/33/45/52/58 in the participants who were seronegative for HPV types at baseline. Figure S2: IgG antibody titers at months 3 and 7 for HPV types 6/11/16/18/31/33/45/52/58 in the participants who were seronegative for HPV types at baseline.

## Abbreviations

The following abbreviations are used in this manuscript:

**Abbreviation**	**Full Term**
AE	adverse event
ALT	alanine aminotransferase
AR	adverse reaction
APTT	activated partial thromboplastin time
AST	aspartate aminotransferase
CI	confidential interval
CREA	creatinine
*E. coli*	*Escherichia coli*
ELISA	enzyme-linked immunosorbent assay
FAS	full analysis set
GMI	geometric mean increase
GMT	geometric mean titer
GST	glutathione-S-transferase
HBG	hemoglobin
HPV	human papillomavirus
IgG	immunoglobulin G
nAb	neutralizing antibody
NMPA	National Medical Products Administration
NIFDC	China National Institute for Food and Drug Control
PBNA	pseudovirion-based neutralization assay
PPS	per-protocol set
PT	prothrombin time
SAE	serious adverse event
sIgA	secretory immunoglobulin A
SS	safety set
VLP	virus-like particle
WBC	white blood cell count
WHO	World Health Organization
