# Supplementary material for: Safety, Tolerability, and Immunogenicity of a Recombinant Nonavalent Human Papillomavirus Vaccine (Escherichia coli) in Healthy Chinese Women Aged 18–45 Years: A Phase 1 Clinical Trial
Source: Vaccines (Basel). 2025 May 13;13(5):511. doi: 10.3390/vaccines13050511 (PMC12115934; doi:10.3390/vaccines13050511)
Supplement: Supplementary file 1 [file vaccines-13-00511-s001.zip › vaccines-3589134-supplementary.pdf]

Supplementary Data

Full list of the inclusion and exclusion criteria:

Inclusion Criteria:

Stage 1:

1. Female subjects 27-45 years of age (inclusive of 27 and 45 years of age);
2. Subjects decided to be healthy by the principal investigator in accordance with such trial subjects' medical history and physical examination results;
3. Subjects who understand and agree to comply with the study procedures and provide written informed consent;
4. Subjects who are able to comply with protocol-specified requirements;
5. Subjects with negative urine pregnancy test result at screening;
6. Subjects with no childbearing potential (e.g. females who have undergone bilateral tubal ligation, hysterectomy, bilateral oophorectomy, etc.), or subjects with childbearing potential who have agreed to abstain from any sexual activity that could result in pregnancy or practice adequate contraception for at least 28 days prior to the first dose and throughout the study;
7. Subjects with axillary temperature≤37.0℃.

Stage 2:

1. Female subjects 18-26 years of age (inclusive of 18 and 26 years of age);
2. Subjects decided to be healthy by the principal investigator in accordance with such trial subjects' medical history and physical examination results;
3. Subjects who understand and agree to comply with the study procedures and provide written informed consent;
4. Subjects who are able to comply with protocol-specified requirements;
5. Subjects with negative urine pregnancy test result at screening;
6. Subjects with no childbearing potential (e.g. females who have undergone bilateral tubal ligation, hysterectomy, bilateral oophorectomy, etc), or subjects with childbearing potential who have agreed to abstain from any sexual activity that could result in pregnancy or practice adequate contraception for at least 28 days prior to the first dose and throughout the study;
7. Subjects with axillary temperature≤37.0℃.

Exclusion Criteria:

The same exclusion criteria apply to both stage 1 and stage 2.

1. Women who are pregnant or breastfeeding, or planning for pregnancy in the following 7 months;
2. Women who have received other HPV vaccine(s) prior to dose 1 of the interventions;
3. Women who have received an investigational or unregistered drug or vaccine within 28 days prior to the first dose of the interventions, or plan to receive an investigational or unregistered drug or vaccine during the study;
4. Women who have known allergy history or who are allergic to any component of the interventions, such as penicillin and amikacin;
5. Women with a history of severe adverse reactions to previous vaccinations, such as allergies, urticaria, dyspnea, angioneurotic edema, or abdominal pain;
6. Women who have an autoimmune disease or immunodeficiency, are HIV positive, or have primary diseases in vital organs;
7. Women who have asthma that is unstable and requires urgent care, hospitalization and the use of oral or intravenous corticosteroids during the past two years;
8. Women who have diabetes mellitus (type I or II), with the exception of gestational diabetes;
9. Women with a history of thyroidectomy or thyroid diseases that required medical care within the past 12 months.;
10. Women with serious angioedema episodes within the past 3 years or requiring medical care over the past 2 years;
11. Women who have hypertension over 145/95 mm Hg at enrolment despite being treated by medication;
12. Women with coagulation disorders as diagnosed by a doctor (e.g. coagulation factor deficiency, coagulopathy, or platelet disorder) or coagulation difficulty;
13. Women with active malignancy, or treated malignancy for which there is no reasonable assurance of sustained cure, or malignancy that is likely to recur during the study;
14. Women with a history of epilepsy other than epilepsy with febrile seizures under two years of age, epilepsy secondary to alcohol use 3 years prior to alcohol withdrawal, or a singular epileptic seizure not requiring treatment within the past 3 years;
15. Women with the condition of asplenia, functional asplenia or any condition resulting in the absence or removal of the spleen;
16. Women with a history of systematic chemotherapy in the past 5 years, a history of immunosuppressive therapy and cytotoxic therapy, and treatment with inhaled corticosteroids within the past 6 months (with the exception of corticosteroid nasal spray for allergic rhinitis or topical corticosteroids for an acute uncomplicated dermatitis), and women who received blood products in the 3 months prior to vaccination with the interventions;
17. Women who received a live attenuated vaccine during the 28 days prior to vaccination with the interventions; Women who received a subunit or inactivated vaccine, such as pneumococcal vaccine, or underwent antianaphylactic treatment during the 14 days prior to vaccination with the interventions;
18. Women who received a subunit or inactivated vaccine, such as pneumococcal vaccine, or underwent antianaphylactic treatment during the 14 days prior to vaccination with the interventions;
19. Women who are currently on an anti-TB prophylaxis or therapy;

20. Women who had fever (with axillary temperature≥38.0°C) during the 3 days prior to vaccination with the interventions or onset of any acute illness that required the use of antibiotics and antiviral treatment within the past 5 days;
21. Women with psychiatric conditions that preclude compliance with the protocol, or women with past or present psychoses, past or present bipolar disorder that has not been well controlled over the past 2 years, or women who are on medication for psychoses, or women who had suicidal thoughts/tendency in the past 5 years prior to enrolment;
22. Women with any medical, psychological or social conditions, or for occupational reasons or otherwise as judged by the principal investigator, that preclude participation in the study, or compromise a subject's ability to give informed consent.

Table S1. Adverse events within 30 days after each vaccination in women aged 27-45 years

	Medium-dose (N = 20)		High-dose (N = 20)		Total (N = 40)		*P* value
	Events	Participants (%)	Events	Participants (%)	Events	Participants (%)	
Total adverse events	33	13 (65.00)	56	19 (95.00)	89	32 (80.00)	**0.048**
Adverse reactions[a]	31	13 (65.00)	48	16 (80.00)	79	29 (72.5)	0.288
Solicited adverse reactions	31	13 (65.00)	47	16 (80.00)	78	29 (72.5)	0.288
Local solicited adverse reactions	20	9 (45.00)	30	15 (75.00)	50	24 (60.00)	0.053
Systemic solicited adverse reactions	11	6 (30.00)	17	7 (35.00)	28	13 (32.50)	0.736
Unsolicited adverse reactions	0	0	1	1 (5.00)	1	1 (2.50)	>0.999
Solicited adverse events	31	13 (65.00)	47	16 (80.00)	78	29 (72.5)	0.288
Unsolicited adverse events	2	2 (10.00)	9	7 (35.00)	11	9 (22.50)	0.130
Adverse events ⩾ grade 3	0	0	1	1 (5.00)	1	1 (2.50)	>0.999
Serious adverse events	0	0	0	0	0	0	-
Discontinuation due to adverse events	0	0	0	0	0	0	-

[a]Any vaccine-related adverse events. Except for the occurrences of AEs, each subject in each trial group was counted only once per row. *P* values were calculated using Pearson's Chi-squared test or Fisher's exact test.

Table S2. Adverse events within 30 days after each vaccination in women aged 18-26 years

	Low-dose (N = 30)		Medium-dose (N = 30)		High-dose (N = 30)		Control (N = 30)		Total (N = 120)		*P* value
	Events	Participants (%)	Events	Participants (%)	Events	Participants (%)	Events	Participants (%)	Events	Participants (%)	
Total adverse events	112	27 (90.00)	73	27 (90.00)	75	22 (73.33)	63	20 (66.67)	323	96 (80.00)	**0.048**[b]
Adverse reactions[a]	93	27 (90.00)	55	24 (80.00)	63	21 (70.00)	52	17 (56.67)	263	89 (74.17)	**0.023**[c]
Solicited adverse reactions	90	27 (90.00)	51	22 (73.33)	57	20 (66.67)	49	17 (56.67)	247	86 (71.67)	**0.034**[c]
Local solicited adverse reactions	65	25 (83.33)	39	21 (70.00)	42	19 (63.33)	36	16 (53.33)	182	81 (67.50)	0.090
Systemic solicited adverse reactions	25	13 (43.33)	12	9 (30.00)	15	9 (30.00)	13	7 (23.33)	65	38 (31.67)	0.403
Unsolicited adverse reactions	3	3 (10.00)	4	4 (13.33)	6	4 (13.33)	3	2 (6.67)	16	13 (10.83)	0.916
Solicited adverse events	90	27 (90.00)	51	22 (73.33)	62	20 (66.67)	49	17 (56.67)	252	86 (71.67)	**0.034**[c]
Unsolicited adverse events	22	12 (40.00)	22	12 (40.00)	13	7 (23.33)	14	9 (30.00)	71	40 (33.33)	0.440
Adverse events ⩾ grade 3	0	0	0	0	1	1 (3.33)	0	0	1	1 (0.83)	>0.999
Serious adverse events	0	0	0	0	0	0	0	0	0	0	-
Discontinuation due to adverse events	0	0	0	0	0	0	0	0	0	0	-

[a]Any vaccine-related adverse events. [b]After Bonferroni adjustment, no significant differences were observed between the groups. [c]The low-dose group showed a higher incidence than did the control group ($P = 0.004$).

Except for the occurrences of AEs, each subject in each trial group was counted only once per row. *P* values were calculated using Pearson's Chi-squared test or Fisher's exact test. The multiple comparisons were adjusted using the Bonferroni method, with $\alpha_{adjusted} = \alpha/6 = 0.008$. Differences were considered statistically significant if $P < 0.008$.

Table S3. List of adverse reactions grade ≥3 that occurred within 7 days after vaccination

Group	ID	Dose	AR term	Grade	Onset time (days post-vaccination)	Duration (day)	Related to vaccination	Outcome
High-dose	X022	2	Induration	3	4	10	Definitely related	Recovered
High-dose	Y094	3	Fever	3	0	4	Possibly related	Recovered

AR: Adverse reaction.

Table S4. Seroconversion rates of HPV type-specific neutralizing and IgG antibodies at month 3 in the PPS cohort

HPV type	Low-dose		Medium-dose		High-dose		Control		*P* value
	n/N	Seroconversion (%) (95%CI)	n/N	Seroconversion (%) (95%CI)	n/N	Seroconversion (%) (95%CI)	n/N	Seroconversion (%) (95%CI)	
nAb									
HPV6	30/30	100.00 (88.43,100.00)	48/48	100.00 (92.60,100.00)	49/49	100.00 (92.75,100.00)	27/27	100.00 (87.23,100.00)	-
HPV11	30/30	100.00 (88.43,100.00)	48/48	100.00 (92.60,100.00)	49/49	100.00 (92.75,100.00)	27/27	100.00 (87.23,100.00)	-
HPV16	30/30	100.00 (88.43,100.00)	48/48	100.00 (92.60,100.00)	49/49	100.00 (92.75,100.00)	27/27	100.00 (87.23,100.00)	-
HPV18	30/30	100.00 (88.43,100.00)	48/48	100.00 (92.60,100.00)	49/49	100.00 (92.75,100.00)	27/27	100.00 (87.23,100.00)	-
HPV31	30/30	100.00 (88.43,100.00)	48/48	100.00 (92.60,100.00)	49/49	100.00 (92.75,100.00)	27/27	100.00 (87.23,100.00)	-
HPV33	30/30	100.00 (88.43,100.00)	48/48	100.00 (92.60,100.00)	49/49	100.00 (92.75,100.00)	27/27	100.00 (87.23,100.00)	-
HPV45	30/30	100.00 (88.43,100.00)	47/48	97.92 (88.93,99.95)	48/49	97.96 (89.15,99.95)	26/27	96.30 (81.03,99.91)	0.882
HPV52	30/30	100.00 (88.43,100.00)	48/48	100.00 (92.60,100.00)	49/49	100.00 (92.75,100.00)	27/27	100.00 (87.23,100.00)	-
HPV58	30/30	100.00 (88.43,100.00)	46/48	95.83 (85.75,99.49)	48/49	97.96 (89.15,99.95)	24/27	88.89 (70.84,97.65)	0.143
IgG antibody									
HPV6	30/30	100.00 (88.43,100.00)	48/48	100.00 (92.60,100.00)	49/49	100.00 (92.75,100.00)	26/27	96.30 (81.03,99.91)	0.175
HPV11	30/30	100.00 (88.43,100.00)	48/48	100.00 (92.60,100.00)	49/49	100.00 (92.75,100.00)	26/27	96.30 (81.03,99.91)	0.175
HPV16	30/30	100.00 (88.43,100.00)	48/48	100.00 (92.60,100.00)	49/49	100.00 (92.75,100.00)	27/27	100.00 (87.23,100.00)	-
HPV18	30/30	100.00 (88.43,100.00)	48/48	100.00 (92.60,100.00)	49/49	100.00 (92.75,100.00)	27/27	100.00 (87.23,100.00)	-
HPV31	30/30	100.00 (88.43,100.00)	48/48	100.00 (92.60,100.00)	49/49	100.00 (92.75,100.00)	27/27	100.00 (87.23,100.00)	-

HPV type	Low-dose		Medium-dose		High-dose		Control		*P* value
	n/N	Seroconversion (%) (95%CI)	n/N	Seroconversion (%) (95%CI)	n/N	Seroconversion (%) (95%CI)	n/N	Seroconversion (%) (95%CI)	
HPV33	30/30	100.00 (88.43,100.00)	48/48	100.00 (92.60,100.00)	49/49	100.00 (92.75,100.00)	27/27	100.00 (87.23,100.00)	-
HPV45	30/30	100.00 (88.43,100.00)	48/48	100.00 (92.60,100.00)	49/49	100.00 (92.75,100.00)	27/27	100.00 (87.23,100.00)	-
HPV52	30/30	100.00 (88.43,100.00)	48/48	100.00 (92.60,100.00)	49/49	100.00 (92.75,100.00)	27/27	100.00 (87.23,100.00)	-
HPV58	30/30	100.00 (88.43,100.00)	48/48	100.00 (92.60,100.00)	49/49	100.00 (92.75,100.00)	27/27	100.00 (87.23,100.00)	-

PPS: per-protocol set, which included participants who received all three vaccine doses and donated serum samples at months 0, 3, and 7, and had no major deviation of the protocol; nAbs: neutralizing antibodies; Seroconversion was defined as at least a fourfold increase in antibody titers over baseline. *P* values were calculated using Pearson's Chi-squared test or Fisher's exact test.

Table S5. Seroconversion rates of HPV type-specific neutralizing and IgG antibodies at month 7 in the PPS cohort

HPV type	Low-dose		Medium-dose		High-dose		Control		*P* value
	n/N	Seroconversion (%) (95%CI)	n/N	Seroconversion (%) (95%CI)	n/N	Seroconversion (%) (95%CI)	n/N	Seroconversion (%) (95%CI)	
nAb									
HPV6	30/30	100.00 (88.43,100.00)	48/48	100.00 (92.60,100.00)	49/49	100.00 (92.75,100.00)	27/27	100.00 (87.23,100.00)	-
HPV11	30/30	100.00 (88.43,100.00)	48/48	100.00 (92.60,100.00)	49/49	100.00 (92.75,100.00)	27/27	100.00 (87.23,100.00)	-
HPV16	30/30	100.00 (88.43,100.00)	48/48	100.00 (92.60,100.00)	49/49	100.00 (92.75,100.00)	27/27	100.00 (87.23,100.00)	-
HPV18	30/30	100.00 (88.43,100.00)	48/48	100.00 (92.60,100.00)	49/49	100.00 (92.75,100.00)	27/27	100.00 (87.23,100.00)	-
HPV31	30/30	100.00 (88.43,100.00)	48/48	100.00 (92.60,100.00)	48/49	97.96 (89.15,99.95)	27/27	100.00 (87.23,100.00)	>0.999
HPV33	30/30	100.00 (88.43,100.00)	48/48	100.00 (92.60,100.00)	49/49	100.00 (92.75,100.00)	27/27	100.00 (87.23,100.00)	-
HPV45	30/30	100.00 (88.43,100.00)	47/48	97.92 (88.93,99.95)	49/49	100.00 (92.75,100.00)	26/27	96.30 (81.03,99.91)	0.441
HPV52	30/30	100.00 (88.43,100.00)	48/48	100.00 (92.60,100.00)	49/49	100.00 (92.75,100.00)	27/27	100.00 (87.23,100.00)	-
HPV58	30/30	100.00 (88.43,100.00)	48/48	100.00 (92.60,100.00)	48/49	97.96 (89.15,99.95)	27/27	100.00 (87.23,100.00)	>0.999
IgG antibody									
HPV6	30/30	100.00 (88.43,100.00)	48/48	100.00 (92.60,100.00)	49/49	100.00 (92.75,100.00)	27/27	100.00 (87.23,100.00)	-

HPV type	Low-dose		Medium-dose		High-dose		Control		*P* value
	n/N	Seroconversion (%) (95%CI)	n/N	Seroconversion (%) (95%CI)	n/N	Seroconversion (%) (95%CI)	n/N	Seroconversion (%) (95%CI)	
HPV11	30/30	100.00 (88.43,100.00)	48/48	100.00 (92.60,100.00)	49/49	100.00 (92.75,100.00)	27/27	100.00 (87.23,100.00)	-
HPV16	30/30	100.00 (88.43,100.00)	48/48	100.00 (92.60,100.00)	49/49	100.00 (92.75,100.00)	27/27	100.00 (87.23,100.00)	-
HPV18	30/30	100.00 (88.43,100.00)	48/48	100.00 (92.60,100.00)	49/49	100.00 (92.75,100.00)	27/27	100.00 (87.23,100.00)	-
HPV31	30/30	100.00 (88.43,100.00)	48/48	100.00 (92.60,100.00)	49/49	100.00 (92.75,100.00)	27/27	100.00 (87.23,100.00)	-
HPV33	30/30	100.00 (88.43,100.00)	48/48	100.00 (92.60,100.00)	49/49	100.00 (92.75,100.00)	27/27	100.00 (87.23,100.00)	-
HPV45	30/30	100.00 (88.43,100.00)	48/48	100.00 (92.60,100.00)	49/49	100.00 (92.75,100.00)	27/27	100.00 (87.23,100.00)	-
HPV52	30/30	100.00 (88.43,100.00)	48/48	100.00 (92.60,100.00)	49/49	100.00 (92.75,100.00)	27/27	100.00 (87.23,100.00)	-
HPV58	30/30	100.00 (88.43,100.00)	48/48	100.00 (92.60,100.00)	49/49	100.00 (92.75,100.00)	27/27	100.00 (87.23,100.00)	-

PPS: per-protocol set, which included participants who received all three vaccine doses and donated serum samples at months 0, 3, and 7, and had no major deviation of the protocol; nAbs: neutralizing antibodies; Seroconversion was defined as at least a fourfold increase in antibody titers over baseline. *P* values were calculated using Pearson's Chi-squared test or Fisher's exact test.

Table S6. Geometric mean increases of HPV type-specific neutralizing and IgG antibodies at month 3 in the participants who were seronegative for HPV types at baseline

HPV type	Low-dose		Medium-dose		High-dose		Control		*P* value
	N	GMI (95%CI)	N	GMI (95%CI)	N	GMI (95%CI)	N	GMI (95%CI)	
nAb									
HPV6	9	138.03 (113.71,294.58)	14	189.35 (110.69,323.94)	16	165.16 (116.43,234.31)	6	125.96 (56.80,279.31)	0.724
HPV11	9	55.88 (35.96,86.85)	14	56.57 (36.20,88.41)	16	65.46 (49.71,86.20)	6	75.67 (34.42,166.34)	0.756
HPV16	9	172.75 (108.08,276.11)	14	211.04 (122.50,363.58)	16	286.21 (183.53,446.33)	6	278.53 (100.17,774.46)	0.484
HPV18	9	215.56 (137.72,337.39)	14	151.52 (80.49,285.23)	16	224.86 (130.72,386.80)	6	131.28 (35.35,487.56)	0.568
HPV31	9	225.70 (125.87,404.72)	14	186.85 (110.65,315.52)	16	233.42 (146.56,371.77)	6	251.79 (96.08,659.84)	0.868
HPV33	9	111.05 (67.61,182.42)	14	78.31 (49.76,123.23)	16	92.33 (60.68,140.50)	6	35.53 (13.11,96.47)	**0.049**[a]

HPV type	Low-dose		Medium-dose		High-dose		Control		*P* value
	N	GMI (95%CI)	N	GMI (95%CI)	N	GMI (95%CI)	N	GMI (95%CI)	
HPV45	9	78.30 (41.57,147.48)	14	25.95 (13.70,49.15)	16	43.75 (28.18,67.95)	6	26.23 (10.62,64.76)	0.051
HPV52	9	213.17 (140.71,322.93)	14	213.95 (144.20,317.44)	16	255.59 (174.51,374.32)	6	205.44 (112.99,373.52)	0.836
HPV58	9	94.45 (47.64,187.25)	14	61.00 (38.85,95.77)	16	103.78 (65.63,164.08)	6	78.88 (23.90,260.29)	0.410
IgG antibody									
HPV6	29	26.73 (22.16,32.24)	45	25.85 (20.58,32.46)	48	26.57 (22.23,31.74)	24	20.83 (16.10,26.95)	0.434
HPV11	29	29.44 (24.61,35.21)	45	32.22 (25.87,40.11)	48	27.05 (22.09,33.12)	24	24.94 (19.41,32.04)	0.401
HPV16	29	23.13 (19.03,28.11)	45	26.12 (20.57,33.16)	48	23.34 (19.07,28.56)	24	22.25 (17.58,28.18)	0.761
HPV18	29	28.71 (24.62,33.46)	45	35.55 (27.32,46.26)	48	24.86 (20.47,30.20)	24	24.85 (18.99,32.53)	0.138
HPV31	29	21.85 (17.91,26.64)	45	21.99 (17.25,28.05)	48	20.22 (16.63,24.59)	24	18.56 (14.06,24.51)	0.684
HPV33	29	11.85 (9.78,14.35)	45	17.08 (13.38,21.80)	48	15.28 (12.52,18.65)	24	12.03 (8.86,16.34)	0.114
HPV45	29	22.70 (18.39,28.02)	45	20.74 (16.42,26.20)	48	18.36 (14.95,22.55)	24	17.20 (13.78,21.48)	0.394
HPV52	29	39.95 (33.22,48.05)	45	34.99 (28.07,43.61)	48	29.52 (24.38,35.75)	24	26.54 (20.37,34.59)	0.080
HPV58	29	20.88 (15.72,27.74)	45	32.31 (26.16,39.90)	48	30.68 (24.90,37.81)	24	18.26 (13.86,24.06)	**0.002**[b]

[a]After Bonferroni adjustment, no significant differences were observed between the groups. [b]Both the medium-dose and high-dose groups showed significantly higher GMIs than did the control group ($P < 0.008$).

nAbs: neutralizing antibodies; GMI: geometric mean increase. Seroconversion was defined as at least a fourfold increase in antibody titers over baseline. GMI was defined as the geometric mean of the fold increases in antibody titers over baseline. *P* values were calculated using ANOVA or Kruskal-Wallis H test. The multiple comparisons were adjusted using the Bonferroni method, with $\alpha_{adjusted} = \alpha/6 = 0.008$. Differences were considered statistically significant if $P < 0.008$.

Table S7. Geometric mean increases of HPV type-specific neutralizing and IgG antibodies at month 7 in the participants who were seronegative for HPV types at baseline

HPV type	Low-dose		Medium-dose		High-dose		Control		*P* value
	N	GMI (95%CI)	N	GMI (95%CI)	N	GMI (95%CI)	N	GMI (95%CI)	
nAb									

HPV type	Low-dose		Medium-dose		High-dose		Control		*P* value
	N	GMI (95%CI)	N	GMI (95%CI)	N	GMI (95%CI)	N	GMI (95%CI)	
HPV6	9	443.91 (196.26,1004.07)	14	363.04 (212.33,620.73)	16	439.87 (292.65,661.13)	6	212.11 (85.83,524.21)	0.362
HPV11	9	109.38 (50.05,239.06)	14	85.06 (55.94,129.35)	16	84.75 (63.82,112.54)	6	75.03 (41.42,135.91)	0.752
HPV16	9	325.57 (163.27,649.19)	14	294.13 (177.12,499.29)	16	424.38 (271.12,664.28)	6	265.07 (114.73,612.44)	0.412
HPV18	9	548.50 (198.32,1517.01)	14	370.38 (222.53,616.46)	16	635.66 (335.81,1203.26)	6	237.26 (49.79,1130.58)	0.300
HPV31	9	481.10 (241.86,957.00)	14	246.98 (142.47,428.16)	16	349.18 (227.15,536.78)	6	233.65 (88.74,615.20)	0.276
HPV33	9	257.76 (123.75,536.87)	14	124.93 (73.02,213.72)	16	196.32 (118.66,324.78)	6	52.21 (22.04,123.67)	**0.011**[a]
HPV45	9	158.04 (73.09,341.70)	14	53.79 (26.68,108.48)	16	138.10 (83.51,228.38)	6	66.84 (24.68,181.05)	**0.042**[b]
HPV52	9	360.29 (165.43,784.67)	14	326.96 (210.69,507.38)	16	415.88 (283.29,610.53)	6	228.89 (79.42,659.69)	0.514
HPV58	9	509.78 (288.10,902.02)	14	243.30 (131.62,449.74)	16	475.49 (363.48,622.03)	6	204.80 (86.34,485.84)	**0.030**[b]
IgG antibody									
HPV6	29	47.86 (37.83,60.55）	45	71.64 (57.07,89.93)	48	71.81 (58.52,88.12)	24	41.51 (31.53,54.64)	**0.001**[c]
HPV11	29	61.17 (46.93,79.75)	45	98.79 (77.79,125.45)	48	91.51 (74.60,112.25)	24	56.10 (41.97,74.97)	**0.003**[d]
HPV16	29	62.13 (48.98,78.81)	45	83.72 (65.87,106.42)	48	103.28 (83.87,127.18)	24	50.50 (37.70,67.66)	**<0.001**[e]
HPV18	29	57.57 (43.82,75.63)	45	81.81 (63.97,104.63)	48	101.89 (80.77,128.53)	24	47.31 (35.96,62.23)	**<0.001**[e]
HPV31	29	52.65 (41.36,67.03)	45	64.31 (51.43,80.41)	48	82.22 (66.86,101.12)	24	39.83 (29.69,53.42)	**<0.001**[e]
HPV33	29	64.22 (49.15,83.91)	45	90.80 (73.11,112.78)	48	120.41 (99.47,145.75)	24	41.37 (31.16,54.91)	**<0.001**[e]
HPV45	29	51.66 (41.42,64.42)	45	71.82 (55.64,92.70)	48	95.50 (76.93,118.55)	24	42.54 (32.30,56.04)	**<0.001**[e]
HPV52	29	74.71 (58.27,95.80)	45	106.55 (85.35,133.02)	48	124.54 (101.85,152.28)	24	50.73 (37.23,69.12)	**<0.001**[e]
HPV58	29	78.78 (60.83,102.03)	45	120.95 (95.61,153.01)	48	149.60 (122.95,182.02)	24	56.42 (42.32,75.24)	**<0.001**[e]

[a]Both the low-dose and high-dose groups showed significantly higher GMIs than did the control group ($P < 0.008$). [b]After Bonferroni adjustment, no significant differences were observed between the groups. [c]Both the medium-dose and high-dose groups showed significantly higher GMIs than did the control group ($P < 0.008$). [d]The medium-dose group showed a significantly higher GMI than did the low-dose group, while both the medium-dose and high-dose groups showed significantly higher GMIs than did the control group ($P < 0.008$). [e]The high-dose group showed a significantly higher GMI than did the low-dose group, while both the medium-dose and high-dose groups showed significantly higher GMIs than did the control group ($P < 0.008$).

nAbs: neutralizing antibodies; GMI: geometric mean increase. Seroconversion was defined as at least a fourfold increase in antibody titers over baseline. GMI was defined as the geometric mean of the fold increases in antibody titers over baseline. *P* values were calculated using ANOVA or Kruskal-Wallis H test. The multiple comparisons were adjusted using the Bonferroni method, with $\alpha_{adjusted} = \alpha/6 = 0.008$. Differences were considered statistically significant if $P < 0.008$.

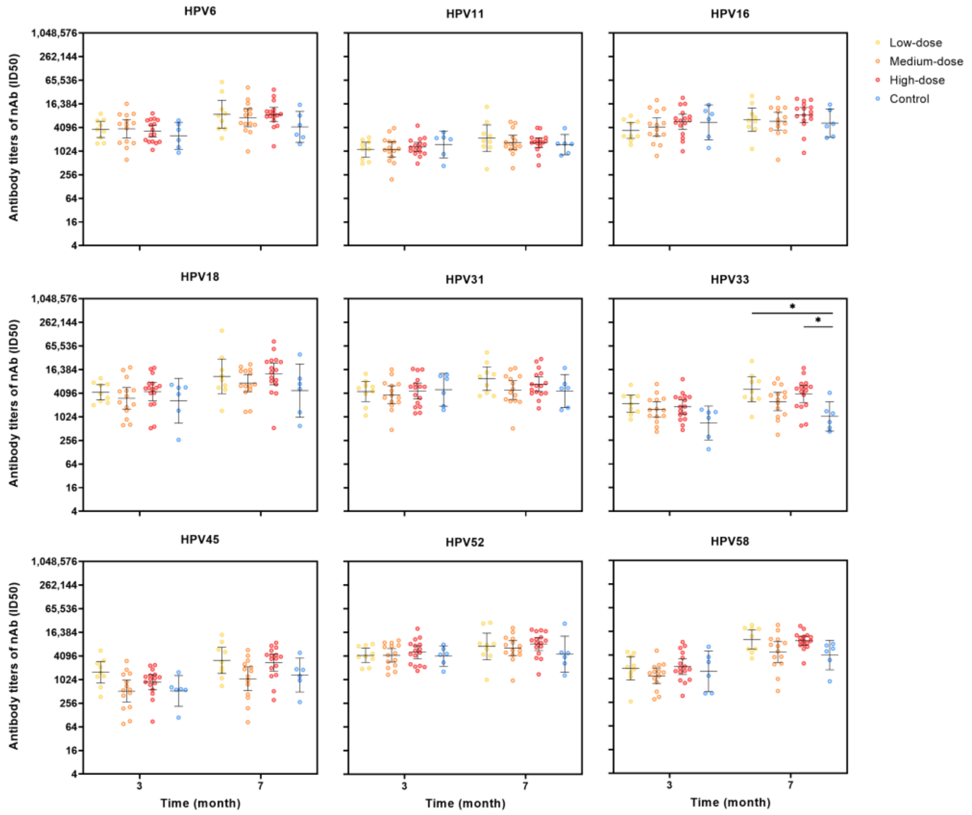

Figure S1. Neutralizing antibodies titers at months 3 and 7 for HPV types 6/11/16/18/31/33/45/52/58 in the participants who were seronegative for HPV types at

baseline. The antibody level of the seronegative sample was artificially set as half of the cutoff value; The black lines indicate the GMTs and 95% CI; CI: confidence interval; GMTs: geometric mean titers; nAb: neutralizing antibody; HPV: human papillomavirus. The multiple comparisons were adjusted using the Bonferroni method, with $\alpha_{adjusted} = \alpha/6 = 0.008$. Differences were considered statistically significant if $P < 0.008$. *The significance is at the $P < 0.008$ level. **The significance is at the P < 0.001 level.

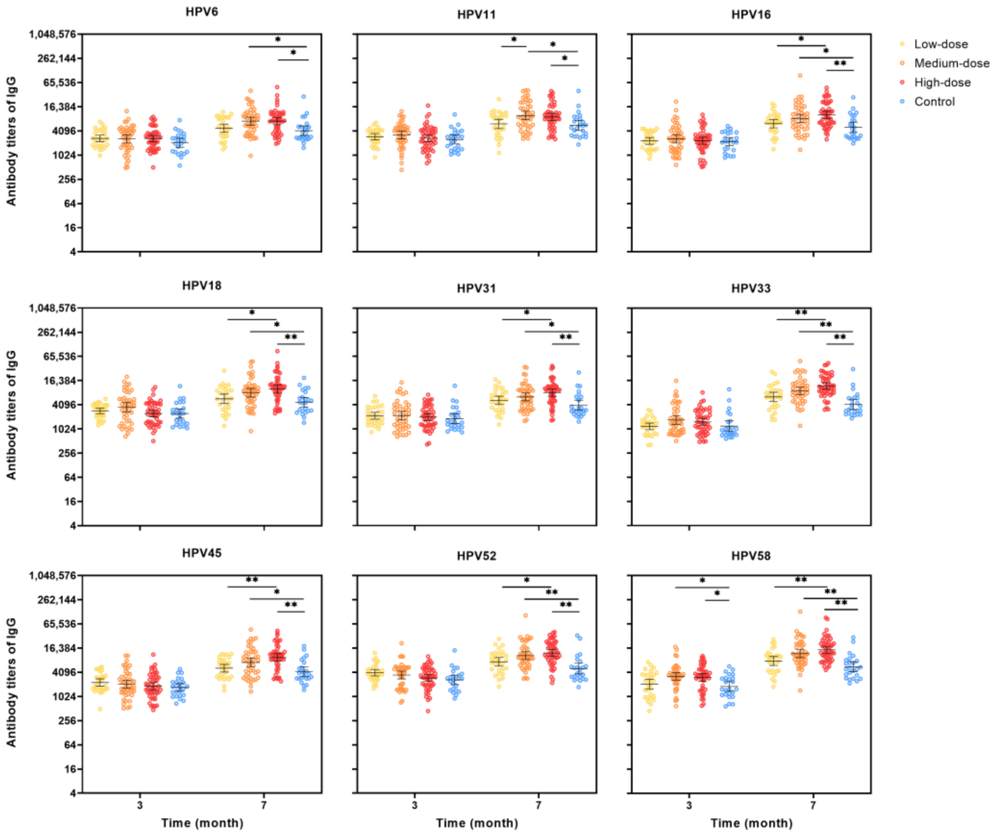

Figure S2. IgG antibodies titers at months 3 and 7 for HPV types 6/11/16/18/31/33/45/52/58 in the participants who were seronegative for HPV types at baseline. The antibody level of the seronegative sample was artificially set as half of the cutoff value; The black lines indicate the GMTs and 95% CI; CI: confidence interval; GMTs: geometric mean titers; HPV: human papillomavirus. The multiple comparisons were adjusted using the Bonferroni method, with $\alpha_{adjusted} = \alpha/6 = 0.008$. Differences were considered statistically significant if $P < 0.008$. *The significance is at the $P < 0.008$ level. **The significance is at the $P < 0.001$ level.
